# Modeling and analysis of flux distributions in the two branches of the phosphotransferase system in *Pseudomonas putida*

**DOI:** 10.1186/1752-0509-6-149

**Published:** 2012-12-06

**Authors:** Andreas Kremling, Katharina Pflüger-Grau, Max Chavarría, Jacek Puchalka, Vitor Martins dos Santos, Víctor de Lorenzo

**Affiliations:** 1 Fachgebiet Systembiotechnologie, Technische Universität München, Garching b. München, Germany; 2 Systems Biology Program, Centro Nacional de Biotecnología-CSIC, Campus Cantoblanco, Madrid, Spain; 3, Helmholtz Center for Infection Research, Braunschweig, Germany; 4, Wageningen University & Research centre, Agrotechnology & Food Sciences, Wageningen, Netherlands; 5, Present address: University Children’s Hospital

**Keywords:** Phosphotransferase System (PTS), Flux Balance Analysis (FBA), Kinetic modelling, Metabolic Control Analysis (MCA)

## Abstract

**Background:**

Signal transduction plays a fundamental role in the understanding of cellular physiology. The bacterial phosphotransferase system (PTS) together with the PEP/pyruvate node in central metabolism represents a signaling unit that acts as a sensory element and measures the activity of the central metabolism. *Pseudomonas putida* possesses two PTS branches, the C-branch (PTS^Fru^) and a second branch (PTS^Ntr^), which communicate with each other by phosphate exchange. Recent experimental results showed a cross talk between the two branches. However, the functional role of the crosstalk remains open.

**Results:**

A mathematical model was set up to describe the available data of the state of phosphorylation of PtsN, one of the PTS proteins, for different environmental conditions and different strain variants. Additionally, data from flux balance analysis was used to determine some of the kinetic parameters of the involved reactions. Based on the calculated and estimated parameters, the flux distribution during growth of the wild type strain on fructose could be determined.

**Conclusion:**

Our calculations show that during growth of the wild type strain on the PTS substrate fructose, the major part of the phosphoryl groups is provided by the second branch of the PTS. This theoretical finding indicates a new role of the second branch of the PTS and will serve as a basis for further experimental studies.

## Background

Mathematical modelling of biological processes is a powerful tool towards the thorough understanding of a biological system. In the mathematical simulation, in the first step, experimental data is reproduced and subsequently, the model can be used to predict the behaviour of the system. This type of iterative model-based analysis is a hallmark of systems biology research that, in the future, is expected to be very helpful in enhancing the understanding of cellular systems in a better way. Here, we chose to analyse the PTS^Ntr^ of *P. putida* with the help of mathematical tools, in order to obtain a functional model that describes the system in detail and may provide new ideas on the physiological role of the PTS^Ntr^ and its cross-talk with the sugar PTS.

*Pseudomonas putida* KT2440 is a ubiquitous Gram-negative, saprophytic soil bacterium, important in biotechnological and systems biological research. This organism is characterized by its metabolic versatility, which enables the strain to use a variety of natural and man-made compounds as carbon and energy source [1], making it an ideal organism to be used in a broad range of biotechnological applications, such as bioremediation or biotransformation processes [2-5].

On one hand, it can degrade degrade a variety of toxic compounds including methoxylated or hydroxylated aromatic acids. On the other hand, *P. putida* is able to degrade fructose [6] and glucose [6-8], two of the most abundant sugars present in plant root exudates [9]. Glucose catabolism occurs in this strain by the simultaneous operation of three peripheral pathways that converge at the level of 6-phosphogluconate, which is then further metabolized via the Entner-Doudoroff pathway [8]. Fructose degradation additionally occurs via the Embden-Meyerhof-Parnas pathway [6].

Central metabolism of *P. putida* is different in comparison to e.g. *E. coli*. However, a common structural metabolic unit that is found in these two organisms, as well as in other bacteria, is the phosphoenolpyruvate-dependent: phosphotransferase system (PTS). In *E. coli* the PTS is the main uptake system for glucose and for other carbohydrates like mannitol, mannose, and N-acetyl-glucosamine. Besides transport properties, the PTS together with the reactions of the central metabolism represents an important signaling unit that measures the glycolytic and gluconeogenetic flux and maps it to the degree of phosphorylation of the PTS proteins [10]. These proteins are involved in a number of signal transduction processes coordinating metabolism and motility of the bacteria [11].

Like many other eubacteria, *P. putida* KT2440 possesses also an alternative PTS, which is encoded by the genes *ptsP*, *ptsN* and *ptsO*. This unusual PTS preserves the phosphotransfer domains but lacks the permease component, ruling out its participation in sugar intake. Because *ptsN* and *ptsO* map adjacently to the *rpoN* gene that encodes nitrogen *σ* factor 54, it was generally believed that this alternative PTS was related to N metabolism. More recent data suggest that this PTS branch predominantly controls the influx of potassium ions [12,13], which can indirectly affect other processes [14]. Furthermore, it could well happen that most reported nitrogen-related phenotypes of PTS-Ntr mutants of *E. coli* could be artifactual [15], thereby raising doubts on the correct designation of the system [16]. While we take notice of current controversy on the name of this PTS branch -and notwithstanding a possible future change in the nomenclature, we will use the name PTS-Ntr [17] for the rest of the article to designate the system composed of the proteins PtsP (EI-Ntr), PtsO (NPr) and PtsN (EIIA-Ntr). Here, we applied a mathematical approach to estimate the flow of the high energy phosphate through the PTS^Ntr^. This was performed in order to get an idea of how the phosphate flow is distributed between the two PTS (PTS^Fru^ and PTS^Ntr^) and to shed some light on the role of both systems in the process of fructose uptake.

Mathematical modeling of the *E. coli* PTS has progressed in the last decade and several models are currently available (reviewed in [10,11]). The models provide evidence that - depending on the type of transport - a clear relationship between the specific growth rate and the degree of phosphorylation of the PTS proteins is valid for a broad range of specific growth rates. Moreover, it could be shown, that the PTS together with glycolytic reactions represents a robust structure in *E. coli*[18]. These results suggest a general principle of cellular control by the PTS that should also be found in other bacteria. Therefore, we set out to analyze in detail the PTS of *P. putida* by mathematically modeling the degree of phosphorylation of its proteins. Since steady-state conditions for the intracellular metabolites (e.g. during a (more or less) constant specific growth rate) contain little information on kinetic parameters, experimental data from a perturbation experiment was used. In these experiments exponential growth of *P. putida* on casamino acids (CAA) was compared with growth on CAA plus fructose. Since uptake of fructose takes place in the late exponential phase [19], this can be seen as a small perturbation and results in a different intracellular steady-state. In a first step - based on data for the wild type strain and mutant strains - a mathematical model was developed that links two input parameters, namely the PEP/pyruvate ratio and the fructose uptake rate to the output, the degree of phosphorylation of one PTS protein, namely PtsN. With the data available it was possible to estimate all equilibrium constants for the biochemical reactions. Based on these initial findings the behavior of the system was predicted for different conditions and the results could be verified in new experiments. In a second step, for both environmental conditions (CAA and CAA plus fructose), flux balance analysis (FBA) was performed based on a stoichiometric model that was introduced previously [20]. Combining results from the PTS model and FBA, new insights into the kinetic properties of the involved enzymes were found. Specifically, we found that the metabolic PEP/pyruvate ratio is faithfully reflected in the phosphorylation state of PtsN, thereby suggesting a mechanism by which the gross physiological state is translated into multiple downstream consequences.

### Biological background

**Figure 1:**
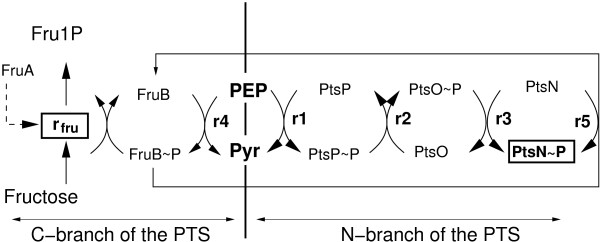
**The PTS reactions in *P. putida*.** The C branch of the PTS is shown on the left hand side from PEP (reaction *r*_4_) while the second branch is shown on the right hand side (reactions *r*_1_−*r*_3_). The phosphryl group from the final protein in the second branch, PtsN is not metabolized. However, there is evidence that cross talk occurs between the two branches. The C branch transfers the phosphryl group to the second branch directly to PtsN (reaction *r*_5_).

*Pseudomonas putida* possesses two distinct phosphoenolpyruvate (PEP) phosphotransferase systems. One is responsible for fructose uptake (carbohydrate branch, PTS^Fru^) and the other is suggested to be involved in signal transduction. In both branches, a high-energy phosphate from PEP is transferred by a number of proteins either to the incoming fructose or to PtsN the last protein in the second branch. As depicted in Figure 1, cross talk may occur and the phosphoryl group from the C branch is transferred to the second branch. Growth of the wild type and three isogenic mutants in the corresponding *pts* genes of *P. putida* in minimal media with different C sources (casamino acids (CAA), CAA plus fructose and CAA plus glucose) revealed different degree of phosphorylation of PtsN. The figure also shows the reactions considered in the mathematical model.

**Figure 2:**
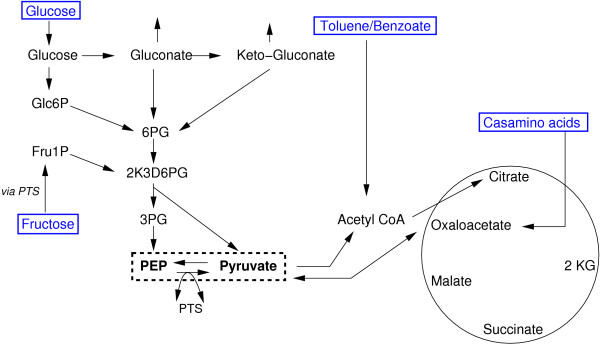
**Central metabolic reactions of *P. putida*.** PEP and pyruvate are hubs that distribute the fluxes to other parts of the network.

By *in vivo* phosphorylation studies using dielectric breakdown and subsequent Western blotting, the direction of the flow of the high-energy phosphate within the PTS^Ntr^ was recently confirmed to succeed in the direction PEP → PtsP → PtsO → PtsN in analogy to the sugar PTS [19,21]. Furthermore, it was shown that both PTS systems do not proceed strictly separated from each other, but that the PTS^Ntr^ and the PTS^Fru^ communicate with each other by phosphate exchange under specific metabolic conditions [22]. Phosphorylation of PtsN by PTS^Fru^ was restricted to conditions in which the PTS^Fru^ was active [22], i.e. it only occurred when cells were grown on fructose, but not on carbon sources that enter the central carbon degradation pathway below the level of glycolysis, like succinate. It is assumed that the FruB protein delivers the phosphate moiety directly to PtsN via its HPr domain, as phosphorylation could also be shown in a *ptsO* mutant strain [22]. Thus, via this cross talk information about the nature of the degraded sugar or the degradation pathway is integrated directly into the phosphorylation state of PtsN. On the other hand it was shown recently that not only the carbon source, but also other physiological conditions, as the nitrogen source, or growth stage influences phosphorylation of PtsN [19]. Monitoring the phosphorylation state of PtsN in the presence of different carbon or nitrogen sources at different growth stages revealed that phosphorylated PtsN was detectable at all growth stages and strongly accumulated in the stationary growth phase, while the non-phosphorylated form exclusively appeared during rapid growth. However, non-phosphorylated PtsN did not disappear at the same growth stage in every case. Instead, the decline of non-phosphorylated PtsN happened earlier in the presence of fructose than in the presence of glucose and earlier in the presence of nitrate than with ammonia. Therefore, it is assumed that the phosphorylation state of PtsN reflects the physiological status of the cell [19]. The catabolic reaction network in *P. putida* is slightly different from *E. coli*, e.g. glucose is metabolized a different way. Figure 2 shows the main reactions including several uptake systems. The central nodes are PEP and pyruvate that serve as a “turning device” that distributes all incoming fluxes, irrespective from their source to their specific metabolic modules. Carbohydrates as well as aromatic carboxylic acids like benzoate feed in at various nodes. The results from FBA (see below) suggest that the main input node from casamino acid uptake is oxaloacetate.

## Methods

### Model for the phosphotransferase system

For the involved PTS proteins shown in Figure 1 the respective differential equations are as follows (only the phosphorylated components are considered):

(1) $$\dot{\mathrm{Pts}P^{P}}\;\;=\;\;r_{1}\;\;-\;\;r_{2}$$

(2) $$\dot{\mathrm{Pts}O^{P}}\;\;=\;\;r_{2}\;\;-\;\;r_{3}$$

(3) $$\dot{\mathrm{Pts}N^{P}}\;\;=\;\;r_{3}\;\;+\;\;r_{5}$$

(4) $$\dot{\mathrm{Fru}B^{P}}\;\;=\;\;r_{4}\;\;-\;\;r_{5}\;\;-r_{\mathrm{fru}}$$

Reactions *r*_1_ to *r*_3_ describe the phosphoryl transfer from PEP via PtsP and PtsO to PtsN, reaction *r*_4_ describes the phosphoryl transfer needed for fructose uptake, and reaction *r*_5_ describes the cross talk between the two branches. A general form of mass action kinetics for the reaction rate is used. The *r*_*i*_ read:

(5) $$r_{i}=k_{i}\left(X^{P}\;\;\;Y\;\;-\;\;K_{i}\;\;X\;\;\;Y^{P}\right)\;\;.$$

where *X* (e.g. *PtsP*) is the phosphoryl donor, *X*^*P*^ the phosphorylated form (e.g. *PtsP*^*P*^) and *Y *, *Y*^*P*^ are the unphosphorylated and the phosphorylated recipient (e.g. *PtsO*, *PtsO*^*P*^). Conservation relations can be taken into account for the four proteins:

(6) $$\mathrm{Pts}P_{0}\;\;=\;\;\mathrm{Pts}P^{P}\;\;+\;\;\mathrm{PtsP}$$

(7) $$\mathrm{Pts}O_{0}\;\;=\;\;\mathrm{Pts}O^{P}\;\;+\;\;\mathrm{PtsO}$$

(8) $$\mathrm{Pts}N_{0}\;\;=\;\;\mathrm{Pts}N^{P}\;\;+\;\;\mathrm{PtsN}$$

(9) $$\mathrm{Fru}B_{0}\;\;=\;\;\mathrm{Fru}B^{P}\;\;+\;\;\mathrm{FruB}$$

The system can be described with five equilibrium constants *K*_*i*_, five velocity reactions *k*_*i*_ and constant entities representing the overall concentration of the proteins.

Scaling is a well known tool to reduce the number of parameters. Scaling on the overall concentration *X*_*i*,0_ of the respective compound and on a chosen time constant leads to a set of equations with seven velocity constants, five equilibrium constants *K*_*i*_ and a scaled uptake rate $\left(r_{\mathrm{fru}}^{\prime }\right)$. System inputs are the PEP/pyruvate ratio and the fructose uptake rate. The respective equations are given in the Additional file 1. Matlab-files to simulate the system are available on request.

### Model for flux balance analysis

The flux distributions were computed by applying Flux Balance Analysis (FBA) on the genome-scale metabolic reconstruction of *P. putida* metabolism iJP815 [20]. Cellular networks are characterized by the observation that there are more unknown reaction rates than equations (for every compound a mass balance equation can be set up). To circumvent this problem an objective function is defined and from all solutions that are admissible the one that maximizes the objective function is chosen. In many cases the maximization of the yield or the maximization of ATP generating fluxes are a good choice, however, different objective functions are possible as well and are analyzed in a systematic way [23]. The following procedure was applied here: The experimentally measured specific growth rates and uptake rate of fructose constituted the constraints of the reconstruction. First, the flux distribution for bacteria growing solely on fructose was identified by setting the specific growth rate to the experimentally measured value and minimizing the uptake rate of fructose. In the first round, however, the experimentally measured uptake rate could not be reached, due to the values of the maintenance parameters. In order to cope with this problem the Non-Growth Associated Maintenance (NGAM) parameter was decreased, so as to obtain the experimentally measured value. The same NGAM value was used for the computations for the remaining conditions (CAA+fructose, CAA). In both cases the flux distributions were ascertained by limiting the specific growth rate and the fructose uptake (where applicable) to the experimentally measured values and minimizing the uptake of CAAs. The latter was achieved by introducing a virtual compound “carbon” along with a set of reactions, each converting “carbon” into a particular amino acid with the stoichiometry defined by the number of carbon atoms possessed by the particular amino acid, and, subsequently, minimizing the amount of carbon needed to be fed into the system. Flux variability analysis (FVA) was performed to show that the optimal flux distribution has narrow ranges and that alternate optima with a complete different pattern can be excluded.

### Kinetics

**Figure 3:**
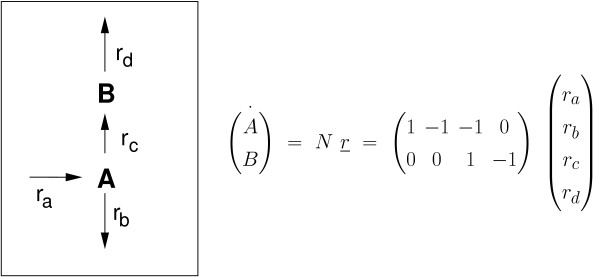
**Network with two metabolites and four reactions.** Given is also the stoichiometric matrix.

Based on the experimental data [19,22], flux distributions for different growth conditions are available that fulfill the steady state conditions. Information on basic kinetic parameters can be obtained with the following approach: Metabolic control analysis is applied to identify rate parameters that, in principle, can be estimated. This is illustrated with the following simple network with two metabolites and four reactions given in Figure 3. To determine all fluxes at least two fluxes have to be measured. This results in two sub-matrices with unknown rates (*N*_*u*_) and known rates (*N*_*kn*_). Introducing kinetic rate laws allows the calculation of the respective elasticities *∈;*_*ij*_=*∂r*_*i*_/*∂c*_*j*_. If it is assumed that the two input fluxes are not independent, but are related by a factor (an assumption that is reasonable while taking into account that for higher uptake rates (e.g. *r*_*a*_) the demands of the cell are changing (e.g. *r*_*b*_)), the following condition holds true for the concentration control coefficients *dc*_*j*_/*dr*_*i*_:

(10) $$\mathrm{\in ;}\;\;\frac{\mathrm{dc}}{\mathrm{du}}\;\;=\;\;-N_{u}^{-1}\;\;N_{\mathrm{kn}}\;\;\underline{\beta }$$

with $\left(\underline{\beta }\right)$ the derivative of the known rates with respect to the chosen input flux *u*, and *∈;* is the matrix with all elasticities. To determine the concentration control coefficients matrix, matrix *∈;* has to be invertible. This requires at least two entries different from zero. Since specific entries of matrix *∈;* are usually not known, an inversion of the matrix *∈;* is only possible if it has the maximal structural rank. To determine the structural rank, we refer to the following definition [24]: “The structural rank (s-Rank) of a structural matrix is the maximum rank a linear matrix of this structure can have.” Therefore, it can be determined by finding the maximum size set of nonzero entries that do not share a row or column of the matrix. If *r*_*a*_ and *r*_*d*_ are the measured rates, and assuming that all remaining reactions *r*_*b*_(*Pyr*), *r*_*c*_(*Pyr*) are irreversible, matrix *∈;* is given by:

(11) $$\mathrm{\in ;}\;\;=\;\;\left[\begin{array}{cc} {}* & 0\\ {}* & 0 \end{array},\;\right]$$

where stars mark the respective dependency. The matrix has structural rank one. In case of *r*_*a*_ and *r*_*b*_ are measured, the respective matrix can be inverted in (nearly) all cases since it is of the form

(12) $$\mathrm{\in ;}\;\;=\;\;\left[\begin{array}{cc} {}* & 0\\ 0 & * \end{array},\;\right]\;.$$

Having the concentration control coefficients at hand, they are used to determine the kinetic properties of the system.

### Strains and experimental conditions

#### Strains, media, and growth conditions

All *Pseudomonas* strains used in this work were derived from strain *Pseudomonas putida* MAD2. *P. putida* MAD2 variants bear directed chromosomal insertions of each gene (*ptsP, ptsO, ptsN, fruB*), with either a kanamycin (*Km*) resistance gene or the *xylE* marker and all mutants used (*ptsP, ptsO, ptsN, fruB*) in this work have been described previously [25,26]. For determination of physiological parameters, bacteria were grown at 30°C in 250 ml baffled shake flasks with 50 ml synthetic M9 medium [27] with 0.2% CAA and 0.2% glucose or fructose as C-source.

#### Analytical procedures and physiological parameters

Cell growth was monitored spectrophotometrically at 600 nm (OD_600_) and fructose/ glucose concentrations were determined enzymatically with the fructose/ glucose assay kit (Sigma-Aldrich) according to the supplier’s manual. The following physiological parameters were determined by regression analysis during the exponential growth phase in batch culture, as described elsewhere [28,29]: maximal specific growth rate, biomass yield on glucose (or fructose), specific glucose (or fructose) consumption. The correlations factors (*rc*) between cellular dry weight (CDW) and OD_600_ were determined from batch cultures of each mutant. Therefore, CDW was measured from at least three parallel 10 ml cell suspensions by harvesting the cells by fast filtration through pre-weight nitrocellulose filters (0.45 *μ*m), which were subsequently washed with 0.9% NaCl and dried at 105°C for 24 h to a constant weight. We have followed the procedures described in [30-32].

#### Determination of the phosphorylation state of PtsN

The phosphorylation state of PtsN in *P. putida* Mad2 and *pts* mutants grown on CAA and glucose was determined by dielectric breakdown and Western blotting as described elsewhere [22].

## Results and discussion

### The PTS model describes the available data points

**Figure 4:**
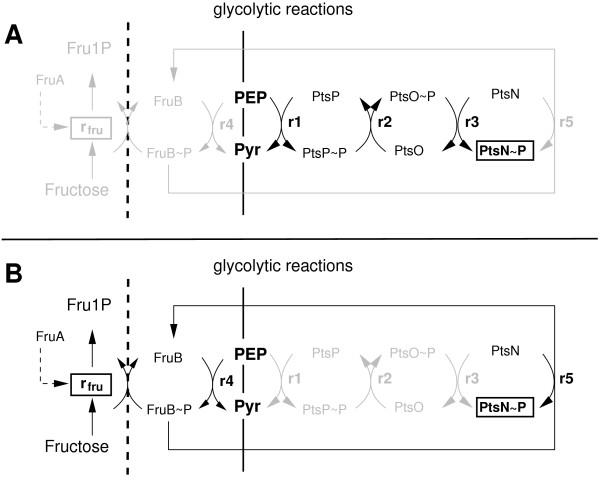
**Network fluxes under different conditions (black lines indicate active fluxes). A** The network for the wild type strain growing on CAA as well as FruB mutant growing on CAA plus fructose. **B** The network of PtsP and PtsO mutants growing on CAA and fructose.

Since metabolic reactions are faster than the growth rate, a pseudo-steady-state can be considered. For the complete model the number of data points (see section Methods) are not sufficient to determine all kinetic parameters directly. However, using the data for the mutant strains allows to decompose the system in a smaller set of equations. Table 1 summarizes restrictions for all reaction rates *r*_*i*_ for the different conditions. The following procedure was applied: The PEP/pyruvate ratio for growth only on CAA was set to 1. As can be seen in Figure 4, mutation in the respective genes of the system prevents the flow of the phosphoryl groups and allows to formulate conditions for some of the kinetic parameters (details for the calculation of the parameters are given in the Additional file 1). Furthermore, the complete system contains a cycle. This means that the second branch and the C branch of the PTS have the same overall equilibrium constant:

(13) $$K_{1}\;\;K_{2}\;\;K_{3}\;\;=\;\;K_{4}\;\;K_{5}\;,$$

**Table 1:** Summary of the restrictions for the determination of the kinetic parameters

**Strain**	**WT**	**PtsP**	**PtsO**	**FruB**
		**(*r***_**1**_**=*r***_**2**_**=0)**	**(*r***_**2**_**=*r***_**3**_**=0)**	**(*r***_**4**_**=*r***_**5**_**=0)**
CAA	*r*_1_=*r*_2_=*r*_3_=0	*r*_3_=*r*_4_=*r*_5_=0	*r*_1_=*r*_4_=*r*_5_=0	*r*_1_=*r*_2_=*r*_3_=0
(*r*_*up*_=0)				
CAA + Fru	–	*r*_3_=*r*_5_=0	*r*_1_=*r*_5_=0	*r*_1_=*r*_2_=*r*_3_=0

The conditions that results directly from the mutation are given in the headline. The conditions that results directly from the medium are given in the respective rows.

providing an additional constraint for the parameters.

**Figure 5:**
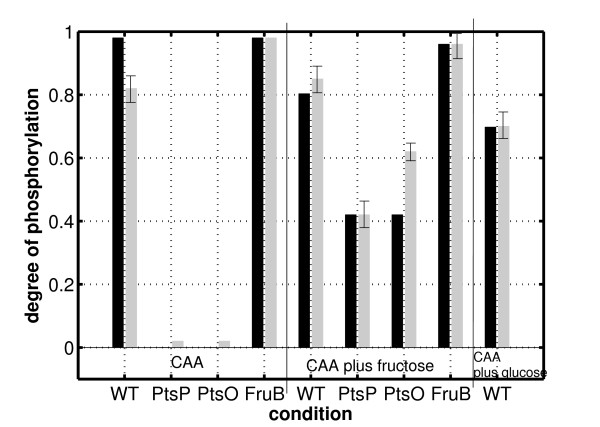
**Comparison of the experimental data with the simulated data.** Growth conditions as indicated on the x-axis. Black bars represent the simulation results while the grey ones the experimental data. Given is the degree of phosphorylation of PtsN. Kinetic parameters are summarized in the Additional file 1 .

Figure 5 shows an comparison of the measured values and the simulation results. Shown are two bars for every condition tested: growth on CAA (4 strains), growth on CAA plus fructose (4 strains), and growth on CAA plus glucose (only wild type). For growth on CAA it assumed that the fructose operon is not induced, that is, the value for the overall concentration of FruB is set to a low value.

The degree of phosphorylation of PtsN of the mutant strains PtsP and PtsO on fructose is characterized by the cross talk between the C and the second branch of the PTS. Although the phosphoryl flux in these mutants is interrupted in the second branch, a certain degree of phosphorylation of PtsN is detected. This can only be explained with a transfer of the phosphoryl group from FruB to PtsN (reaction *r*_5_). In contrast, for growth on CAA, it is assumed that the *fru* operon is not induced and the levels of phosphorylation for both mutants are low. The PEP pyruvate ratio was estimated to:

(14) $$pp_{\mathrm{CAA}}=1.0\;\;>\;\;pp_{\mathrm{CAA}+\mathrm{Fru}}=0.49\;.$$

For growth on CAA plus glucose, the values for *pp*_*CAA* + *Glc*_ and the concentration of FruB represent degrees of freedom and have to be adjusted to describe the data. From the available data on the degree of phosphorylation, FruB is assumed to be present also on growth on glucose, however, it has to be further analyzed whether this is a specific effect of glucose or an artefact of residual presence of fructose in the medium composition.

### Model prediciton and verification

**Figure 6:**
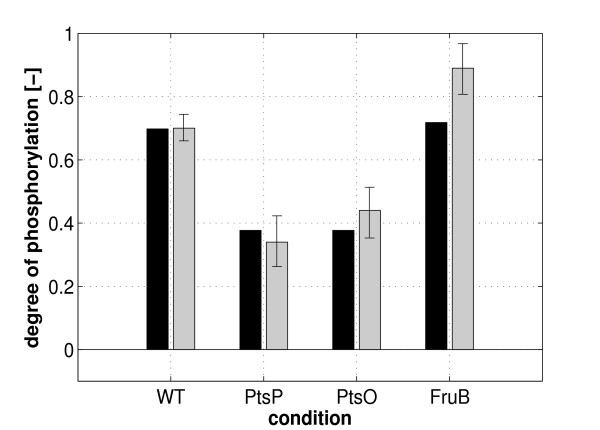
**Comparison of the experimental data with the predicted simulated data for growth in glucose.** Black bars represent the simulation results while grey bars are the experimental data. Given is the degree of phosphorylation of PtsN.

To check the performance of the model, the behavior of the model for growth of *P. putida* under different environmental conditions was predicted. Here, growth of mutant strains PtsP, PtsO, and FruB on glucose was predicted and finally verified experimentally. Table 2 summarizes the respective values. Figure 6 shows the comparison of the simulation results with the new experimental data. For the PtsP and PtsO mutant, again, the cross talk between the C and the second branch of the PTS can be seen. The model predicts the values for all conditions very well.

**Table 2:** Degree of phosphorylation of PtsN grown on CAA plus glucose

	**WT**^**a**^	**PtsP**	**PtsO**	**FruB**
**PtsN/PtsN**_***0***_	**0.7*±0.03***	**0.34*±0.02***	**0.44*±0.08***	**0.89*±0.08***


^a^Value already published.

### Flux distributions at nodes PEP and pyruvate

FBA is widely used to explore the capabilities of genome-scale networks (for a review, see [33]). However, it was also shown that FBA can be used to estimate flux distributions in cellular networks. In particular, for the genome scale model available for *P. putida* it could be shown, that there is a good agreement between the calculated values and measured values [20].

**Figure 7:**
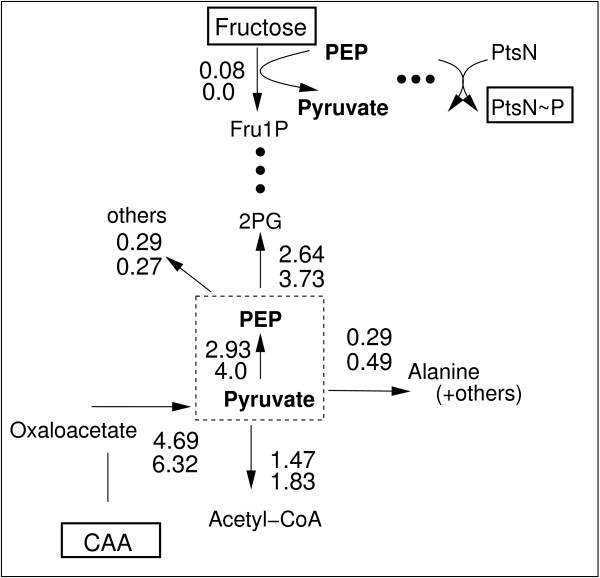
**Flux map at node PEP pyruvate for growth conditions of the wild type on CAA plus fructose (upper values) and CAA only (lower values).** The ratio of PEP and pyruvate determines the degree of phosphorylation of PtsN.

FBA can be used to estimate flux distributions given a stoichiometric network, uptake and production rates for compounds in the medium and an objective function. Here, the network was analyzed for growth on CAA and growth on CAA plus fructose. The complete flux distribution can be found in the Additional file 2. Figure 7 highlights the flux distributions at nodes PEP and pyruvate. Shown are the values for growth on CAA plus fructose (upper value) and for CAA only (lower value). The number of reactions where either PEP or pyruvate or both are involved for these conditions is 15. The main part of the fluxes is coming via oxaloacetate to the node pyruvate and is then further distributed to acetyl CoA and PEP. Minor drain fluxes are directed to alanine and other compounds. Note that in the stoichiometric network only the metabolic part is considered (C branch) but not the detailed reactions for the second branch of the PTS. To check the results for alternate optima, FVA was performed and the respective minimal and maximal values are computed. Table 3 summarizes the values for the fluxes where PEP and pyruvate are involved. As can bee seen, the minimal and maximal values show only a small range. The deviation between minimal and maximal values based on the nominal value is smaller than 3% except for the drain from pyruvate to acetyl CoA. This indicates that alternate optima with a complete different flux distribution could be excluded.

**Table 3:** Summary of FVA

**Substrate**	**Oxaloacetat.**	**pyruvate to**	**pyruvate**	**PEP to 2PG**
	**to pyruvate**	**acetylCoA**	**to PEP**	
**CAA** - FBA	6.32	1.83	4.00	3.73
FVA min/max	6.18/6.35	1.68/1.85	3.96/4.01	3.68/3.78
(max-min)/nominal	2.7%	9.3%	1.3%	2.7%
**CAA + Fru** - FBA	4.69	1.47	2.93	2.64
FVA min/max	4.59/4.71	1.36/1.49	2.90/2.97	2.61/2.68
(max-min)/nominal	2.6%	8.8%	2.4%	2.7%

Given are the results of FBA for the main fluxes with PEP or pyruvate as substrate or product (first row) with the respective minimal and maximal values (second row)The third row gives the ratio (max-min)/nominal value (in per cent).

### Kinetic properties at PEP and pyruvate node

**Figure 8:**
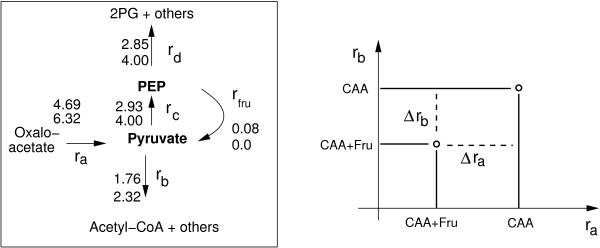
**Left: Reduced flux map at node PEP/pyruvate.** The network is represented with 5 reactions rates and 2 nodes. Right: An increasing input flux *r*_*a*_ leads to increasing other fluxes. The increase in flux *r*_*b*_ is calculated with the slope $\left(k=\frac{\Delta r_{b}}{\Delta r_{a}}\right)$ .

Having the results from the previous sections at hand, a mathematical model taking into account the kinetics of reactions where PEP and pyruvate are involved was set up. The focus is on the steady-state values of intracellular metabolites PEP and pyruvate in dependence on the input flux. Therefore, fluxes that produce PEP or pyruvate or have PEP or pyruvate as substrate were summed up. This resulted in a network with 5 fluxes and two nodes as shown in Figure 8 on the left side. Based on the structure of the network, the approach introduced above (Figure 3), and the FBA results, kinetic expressions were set up for reactions *r*_*c*_ and *r*_*d*_, since in this case matrix *∈;* has full rank. To perform the calculation for different input fluxes *r*_*a*_, the values for *r*_*b*_ had to be interpolated as shown in Figure 8 to estimate a factor *k* (the relation between the two fluxes, see Methods). A linear interpolation was used and the values for *r*_*b*_ are given as follows:

(15) $$r_{b}\;\;=\;\;r_{b}^{s}\;\;+\;\;k\;(r_{a}-r_{a}^{s})$$

with $\left(r_{a}^{s}\right)$ and $\left(r_{b}^{s}\right)$ are the respective values for growth in CAA plus fructose and *k* is the slope as shown in Figure 8. For the following considerations, the very low PTS flux is not considered; this results in the following steady-state conditions:

(16) $$\begin{array}{c} \left(\begin{array}{c} \dot{\mathrm{PEP}}\\ \dot{\mathrm{Prv}} \end{array}\right)\;=\;\left(\begin{array}{c} \;0\;\\ \;0\; \end{array},\;\right)\;=\;\left(\begin{array}{cc} 1 & \;-1\\ -1 & \;0 \end{array},\;\right)\;\;\left(\begin{array}{c} r_{c}\\ r_{d} \end{array}\right)\\ \;+\;\;\left(\begin{array}{cc} 0 & \;0\\ 1 & \;-1 \end{array},\;\right)\;\;\left(\begin{array}{c} r_{a}\\ k\;r_{a} \end{array},\;\right)\;. \end{array}$$

The elasticity matrix is in the form:

(17) $$\;\mathrm{\in ;}\;\;=\;\;\left(\begin{array}{cc} 0 & \;{\mathrm{\in ;}}_{c}\\ {\mathrm{\in ;}}_{d} & \;0 \end{array},\;\right)\;\;=\;\;\left(\begin{array}{cc} \;0 & \;\frac{dr_{c}}{\mathrm{dPyr}}\\ \frac{dr_{d}}{\mathrm{dPEP}} & 0 \end{array},\;\right)$$

and the respective concentration control coefficient are:

(18) $$\begin{array}{lll} \frac{\mathrm{dPEP}}{dr_{a}}\;\; & =\;\;\frac{1-k}{{\mathrm{\in ;}}_{d}}\;\\ \frac{\mathrm{dPyr}}{dr_{a}}\;\; & =\;\;\frac{1-k}{{\mathrm{\in ;}}_{c}}\; & \; \end{array}$$

For the unknown reaction rates *r*_*c*_ and *r*_*d*_ power law kinetics are applied:

(19) $$\begin{array}{lll} r_{c}\;\; & =\;\;k_{c}\;\mathrm{Py}r^{n_{c}}\;\\ r_{d}\;\; & =\;\;k_{d}\;\mathrm{PE}P^{n_{d}}\;\; & \; \end{array}$$

and the respective elasticities are

(20) $$\begin{array}{lll} {\mathrm{\in ;}}_{c}\;\; & =\;\;n_{c}\;\frac{r_{c}}{\mathrm{Pyr}}\;\\ {\mathrm{\in ;}}_{d}\;\; & =\;\;n_{d}\;\frac{r_{d}}{\mathrm{PEP}}\;.\; & \; \end{array}$$

From the observation that for increasing input flux *r*_*a*_ the PEP/pyruvate ratio is also increasing, a necessary condition for the positive slope can be calculated from the following condition:

(21) $$\frac{d\frac{\mathrm{PEP}}{\mathrm{Pyr}}}{dr_{a}}\;\;=\;\;\frac{\mathrm{Pyr}\;\frac{\mathrm{dPEP}}{dr_{a}}\;-\;\mathrm{PEP}\;\frac{\mathrm{dPyr}}{dr_{a}}}{\mathrm{Py}r^{2}}\;\;>\;\;0$$

and therefore

(22) $$\frac{\mathrm{dPEP}}{\mathrm{dPyr}}\;\;>\;\;\frac{\mathrm{PEP}}{\mathrm{Pyr}}\;.$$

Inserting Equations (18) and (20) result in:

(23) $$\frac{n_{c}}{n_{d}}\;\;>\;\;1\;,$$

which give first insights for the kinetic parameters. Since for the (relative) simple model, the steady-state values for PEP and pyruvate can be calculated, an estimation of the kinetic parameters is possible. With *r*_*c*_=*r*_*d*_, *αk*_*d*_=*k*_*c*_, and *n*^*′*^=1/*n*_*d*_−1/*n*_*c*_ the PEP/pyruvate ratio is:

(24) $$\frac{\mathrm{PEP}}{\mathrm{Pyr}}\;\;=\;\;\alpha^{1/n_{d}}\;\;{\left(\frac{r_{c}(r_{a})}{k_{c}}\right)}^{\mathrm{n\prime}}\;.$$

Having two measurements available, two values (*α* and *n*^*′*^, corresponding to *k*_*d*_ and *n*_*d*_) could be calculated when *k*_*c*_=1 and *n*_*c*_=1 (this choice is reasonable since we are mainly interested in a comparison of the two enzymes rather than in absolute values).

**Figure 9:**
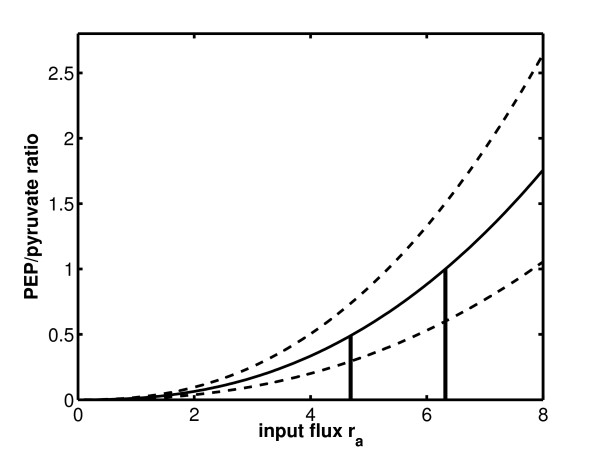
**Characteristic curve for the dependency of the PEP/pyruvate ratio on the input flux *r***_***a***_**.** The two measured data points are indicated with a line.

Figure 9 shows the relation between the PEP/pyrvuate ratio and the input flux.The curve for growth on CAA is shown. In case of growth on CAA plus fructose, the flux through the PTS is very small. Therefore, the curve is nearly identical and was not shown in the figure. Based on the flux values reported in Table 3, value *k* (see Figure 8) might change also which results in a different behavior of the kinetics. In Figure 9 two extreme cases were also simulated (dashed lines) representing the largest and the smalles value of *k*.

### Interrelation between the two branches of the PTS

**Figure 10:**
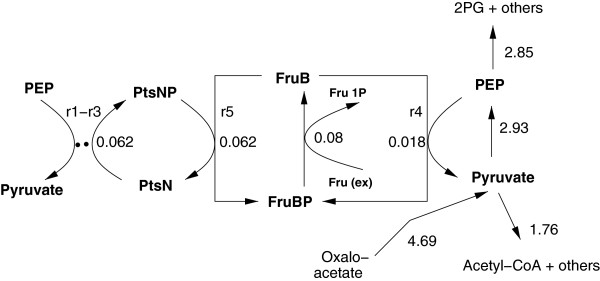
**Complete flux distribution at node PEP and pyruvate for growth on CAA plus fructose.** Note, that the major part for the phosphorylation of incomming fructose comes from the second branch of the PTS.

Having both parts of the model available, an estimation of the complete flux distribution, considering fluxes through both parts of the PTS branches, is possible. Table 1 shows all equilibrium conditions for the individual reaction rates. As can be seen, for the wild type strain and growth on CAA plus fructose, none of the individual reaction is in equilibrium (column WT, row CAA + Fru). Therefore this experiment could not used for the determination of the kinetic parameters. The equation system in this case (representing a steady-state) given in the Additional file 1 (Equation (4)) is valid to determine the individual fluxes given all kinetic parameters. Taking into account that the equations are scaled (as described in the Additional file 1), a recalculation of the fluxes, based on literature values for the intracellular components, leads to the flux distribution shown in Figure 10. The most interesting result is that – under the investigated condition of a small fructose uptake rate – approx. 78% of the required phosphoryl groups for the fructose uptake are provided by the second branch of the PTS. The strength of this coupling is unexpected since the C branch of the PTS was thought to be an autonomous module. Furthermore, there are no theoretical studies with *E. coli* available that show that the second branch of the PTS is involved in carbohydrate transport. The explanation is as follows: The overall equilibrium constant requires that both branches (PEP to PtsN via the C branch, and PEP to PtsN via the second branch) have the same value. From the experimental data, cross talk occurs with a rather low value of the equilibrium constant *K*_5_. This leads to a rather small value for *K*_4_ and corresponding to a high affinity of PEP to FruB. The high affinity leads to a high phosphorylation of FruB, 99%, hence, the substrate, unphosphorylated FruB, is rare and consequently, the flux *r*_4_ is low. This result is rather unexpected and poses new questions on the function of cross talk.

## Conclusion

The PTS is a key player in the coordination of catabolic reactions in *E. coli*[11]. The second branch of this signaling unit recently gained attention [34] since it is involved in the regulation of nitrogen metabolism and carbon assimilation. Moreover, this unit plays also a role in potassium homoeostasis by regulating a potassium transport system [12]. Several mathematical models for the sugar phosphotransferase system in *E. coli* are published [10,18] that reveal a clear relationship between the input flux of carbon and the degree of phosphorylation of the PTS protein EIIA. This was shown by a comparison of experimental data for growth on different PTS and non-PTS sugars. However, kinetic data for the N branch of the system can hardly be found, since measurement of the degree of phosphorylation of the respective proteins “in vivo” are missing. In contrast, for *Pseudomonas putida* experimental data on the phsophorylation state of the PTS protein PtsN is available from a perturbation experiment and this data was used to set up and validate a mathematical model.

The model comprises two parts: The first one relates the PEP/pyruvate ratio for different growth conditions of a wild type strain and mutant strains (PtsP, PtsO, FruB) to the degree of phosphorylation of the PTS protein PtsN. Based on the information for the different mutant strains, the complete set of equations could be simplified for every growth condition/strain and the equilibrium constants were calculated or chosen to describe the experimental data at best. Moreover, it was possible to estimate the PEP/pyruvate ratio for growth on CAA plus fructose if the PEP/pyruvate ratio for growth on CAA alone was given. The experimental results are in good agreement with the simulated data; this is reflected by the fact that the mean of the residuals is in the range of 10% of the measured values (the errors in the experimental data are between 5% and 30%). The experimental data revealed differences in the phosphorylation state of PtsN between the wild type and the FruB mutant strain for growth on CAA as well as for the PtsP and PtsO mutant for growth on fructose. These differences could not be reproduced by the model, since in both cases the mathematical equations are the same. However, it was assumed in the model that the PEP/pyruvate ratio is the same for one substrate. So, the differences could be explained with slightly different values of the PEP/pyruvate ratio for growth on the same substrate. The overall equilibrium constant for the PTS was estimated to be *K*_*eq*_=*K*_1_*K*_2_*K*_3_=*K*_4_*K*_5_=0.02. This value is a factor 35 smaller than reported for the *E. coli* PTS [18]. However, the value used in [18] is based on different studies with enzymes *in vitro* and therefore a fair comparison is not possible. The equilibrium constant for the cross talk between the two branches, parameter *K*_5_, is 654.6, indicating a very weak connection between the two branches.

Based on the kinetic parameters obtained from the experiments with the wild type *P. putida* strain and the mutant strains, the PEP/pyruvate ratio for growth in CAA plus glucose was adjusted to describe the data. Since the degree of phosphorylation of PtsN under this condition is much lower than for growth on CAA or CAA plus fructose, the PEP/pyruvate ratio is an order of magnitude smaller *pp*_*CAA* + *Glc*_=0.05. Based on the data, a prediction of the degree of phosphorylation of PtsN was performed for different mutant strains (PtsP, PtsO, and FruB). The experimental data could be reproduced very well.

Having PEP/pyruvate ratios for growth on CAA and CAA plus fructose available, in a second step, kinetic properties of the system were analyzed based on the respective flux distributions. As expected the flux pattern was different for the two conditions. The main fluxes from or to the both nodes PEP and pyruvate are shown in Figure 7. The uptake of fructose results in a decrease of the main flux to pyruvate and simultaneously in the flux from PEP to the upper part of glycolysis, while other fluxes to alanine and others remain more or less constant. FVA was performed and indicates only small ranges for the minimal and maximal values. This excludes alternate optima with a complete different flux distribution. Taking power law kinetics into consideration for the two rates *r*_*c*_ and *r*_*d*_ and using the other fluxes as input fluxes, conditions for the hill coefficient for both enzymes could be calculated. Furthermore, setting the two parameters for enzyme PEP synthase (*r*_*c*_) to 1 the ratio of the parameters of the enzyme for *r*_*d*_ (representing gluconeogenetic reactions) is calculated. As a result, the hill coefficient for the gluconeogenetic reactions is smaller then the one for PEP synthase (factor 0.30) while the reaction constant *k* is higher (factor 2.73). Taking into account the values from flux variability analysis a confidence region for the kinetics shown in Figure 9 can be given.

An interesting observation was seen when calculating all reaction rates in case of growth on CAA plus fructose. In this case, the individual reactions are not in equilibrium since fructose as additional input enhances the PTS reaction. Approx. 78% of the required phosphoryl groups for the fructose uptake are provided by the second branch of the PTS by cross talk.

This result allows to speculate on a complete new and unexpected role for the PTS^Ntr^, at least in *P. putida*. It might not only be involved in the regulation of various processes, as the activity of central enzymes [35], the accumulation of polyhydroxyalcanoates [29], or the expression of the toluene degradation pathway [25], but serve also as the main provider of phosphoryl groups for fructose uptake. One could speculate that by having the phosphoryl groups cycling in the PTS^Ntr^, they provide a storage system of rapidly available phosphate. This system comes into action when fructose is provided to the cells. Fructose in *P. putida* is degraded by both pathways, the EMP and the ED pathway [36], whereas glucose is degraded almost exclusively via the ED pathway in *P. putida* as the fructose-6-phosphate kinase, able to catalyze the conversion of fructose-6-phsophate to fructose-1,6-biphosphate and thereby making the connection to the EMP pathway, is missing [6]. Degradation of fructose via the EMP pathway is thermodynamically more favourable than degradation via the ED pathway. It is estimated that per molecule fructose degraded through the EMP pathway one molecule ATP is gained compared to degradation following the ED pathway [37]. Thus, the PTS^Ntr^ might serve as a “pre”-adaptation to the potential presence of fructose, enabling the cell to rapidly and efficiently metabolize fructose, when it is available. This hypothesis is currently under investigation in our laboratory.

Modeling of signal transduction units together with genome scale stoichiometric models will help for a better understanding of the cellular system. Especially the PTS is an important system that is involved in the coordination of catabolic reactions. The proposed model is good starting point to extend research in direction of the coordination between the carbon and other networks.

## Competing interest

The authors declare that they have no competing interests.

## Authors’ contributions

AK performed the modeling of the PTS, KPG and MC performed the experiments, JP and VMdS performed the flux balance analysis, AK and VdL designed the study, AK and KPG wrote the manuscript. All authors read and approved the final manuscript.

## Supplementary Material

Additional file 1 The files describes the model equations and the kinetic parameters. Click here for file

Additional file 2 The xls-sheet gives all values for the flux distributions based on a FBA analysis for different growth conditions. The xls-sheet is subdivided into two parts.In the first part, the values for the growth conditions are summarized; afterwards the flux for each reaction for the different conditions is provided. For all conditions, three columns are shown: given are the nominal value, and minimal/maximal values based on FVA. Reactions with PEP and pyruvate as substrate (S) or product (P) are highlighted. Click here for file
