# Supplementary material for: Modeling and analysis of flux distributions in the two branches of the phosphotransferase system in Pseudomonas putida
Source: BMC Syst Biol. 2012 Dec 6;6:149. doi: 10.1186/1752-0509-6-149 (PMC3562155; doi:10.1186/1752-0509-6-149)
Supplement: Additional file 1 — The files describes the model equations and the kinetic parameters. [file 1752-0509-6-149-S1.pdf]

Modeling and analysis of flux distributions in the two branches of the phosphotransferase system in Pseudomonas putida – Supporting information file 1

Andreas Kremling*[1] , Katharina Pflüger-Grau[2], Max Chavarría[2], Jacek Puchalka[3], Vitor Martins dos Santos[4] and Víctor de Lorenzo[2]

[1] Fachgebiet Systembiotechnologie, Technische Universität München [2] Systems Biology Program, Centro Nacional de Biotecnología-CSIC, Campus Cantoblanco, Madrid, Spain [3] Helmholtz Center for Infection Research, Braunschweig, Germany [4] Wageningen University & Research centre, Agrotechnology & Food Sciences, Wageningen, Netherlands

Email: Andreas Kremling*- a.kremling@tum.de;

*Corresponding author

PTS equations and scaling

The complete set of equations for the PTS proteins that are used is given in the following form:

$$
\begin{aligned}
\dot{PtsP^P} &= k_1(PEP\,PtsP \,-\, K_1 Pyr\,PtsP^P) \,-\, k_2(PtsP^P\,PtsO \,-\, K_2\,PtsP\,PtsO^P) \\
\dot{PtsO^P} &= k_2(PtsP^P\,PtsO \,-\, K_2\,PtsP\,PtsO^P) \,-\, k_3(PtsO^P\,PtsN \,-\, K_3\,PtsO\,PtsN^P) \\
\dot{PtsN^P} &= k_3(PtsO^P\,PtsN \,-\, K_3\,PtsO\,PtsN^P) \,+\, k_5(FruB^P\,PtsN \,-\, K_5\,FruB\,PtsN^P) \\
\dot{FruB^P} &= k_4(PEP\,FruB \,-\, K_4 Pyr\,FruBP^P) \,-\, k_5(FruB^P\,PtsN \,-\, K_5\,FruB\,PtsN^P) \\
&\quad - r_{fru}
\end{aligned} \tag{1}
$$

Introducing the conservation equation $X + X^P = X_0$ and scaling with $x = X^P/X_0$, $\tau = k_1\,Pyr$ leads to (prime indicates derivative with respect to τ):

$$
\begin{aligned}
ptsP^{P\prime} &= \frac{PEP}{Pyr}\,(1 - ptsP) \,-\, K_1\,ptsP^P \\
&\quad - \frac{k_2 PtsO_0}{k_1 Pyr}\,(ptsP^P\,(1 - ptsO^P) \,-\, K_2\,ptsP\,ptsO^P)
\end{aligned}
$$

$$
\begin{aligned}
ptsO^{P\prime} &= \frac{k_2 PtsP_0}{k_1 Pyr}\,(ptsP^P\,(1-ptsO^P)\;-\;K_2\,(1-ptsP^P)\,ptsO^P) \\
&\quad -\frac{k_3 PtsN_0}{k_1 Pyr}\,(ptsO^P\,(1-ptsN^P)\;-\;K_3\,(1-ptsO^P)\,ptsN^P) \\
ptsN^{P\prime} &= \frac{k_3 PtsO_0}{k_1 Pyr}\,(ptsO^P\,(1-ptsN^P)\;-\;K_3\,(1-ptsO^P)\,ptsN^P) \\
&\quad +\frac{k_5 FruB_0}{k_1 Pyr}\,(fruB^P\,(1-ptsN^P)\;-\;K_5\,(1-fruB^P)\,ptsN^P) \\
fruB^{P\prime} &= \frac{k_4}{k_1}\,(\frac{PEP}{Pyr}\,(1-fruB^P)\;-\;K_4\,fruBP^P) \\
&\quad -\frac{k_5 PtsN_0}{k_1 Pyr}(fruB^P\,(1-ptsN^P)\;-\;K_5\,(1-fruB^P)\,ptsN^P) \\
&\quad -\frac{r_{fru}}{k_1 Pyr FruB_0} \qquad (2)
\end{aligned}
$$

The dimensionless system has two inputs (PEP/pyruvate ratio and scaled fructose uptake rate), 5 equilibrium constants K_i and seven kinetic parameters α_j:

$$
\begin{aligned}
&\alpha_1 = \frac{k_2 PtsO_0}{k_1 Pyr} \qquad \alpha_2 = \frac{k_2 PtsP_0}{k_1 Pyr} \qquad \alpha_3 = \frac{k_3 PtsO_0}{k_1 Pyr} \\
&\alpha_4 = \frac{k_3 PtsN_0}{k_1 Pyr} \qquad \alpha_5 = \frac{k_5 FruB_0}{k_1 Pyr} \qquad \alpha_6 = \frac{k_4}{k_1} \qquad \alpha_7 = \frac{k_5 PtsN_0}{k_1 Pyr} \qquad (3)
\end{aligned}
$$

Note, that in the most cases considered in the manuscript, a number of rates are zero. In these cases, scaling does not play any role, since the equilibrium is invariant. The most important case for the complete set is growth of the wild type strain on CAA plus fructose. Here, the individual rates are not zero. For the equations for $ptsO^p$ and $ptsN^p$, factors α_2, α_3, α_4 are assumed to be in the same order of magnitude, since all have identical structures. For the equations for $ptsP^p$ and $fruB^p$ different expressions appear. Since the concentration of a metabolite is far higher than the one of a protein, a factor 100 is used here that relates the values for PEP/Pyr and α_2; and α_6 and α_7, respectively. To obtain a scaled value for the uptake rate r_{fru} the following rough estimation was performed: The value of the fructose uptake rate is $r_{fru} = 0.08$ mmol/gDW h. Parameter k_1 is 2 $10^5 1/(\mu$mol/gDW h) [Kremling et al., Analysis of global control of *Escherichia coli* carbohydrate uptake, BMC Systems Biology 42, 2007], the concentration of pyruvate in the order of $10\,\mu$mol/gDW [Sauter and Gilles, Modeling and experimental validation of the signal transduction via the *Escherichia coli* sucrose phosphotransferase system, Journal of Biotechnology 110, 2004; Bettenbrock et al., A quantitative approach to catabolite repression, JBC 281, 2006] and the

concentration of a PTS protein in the order of 1 $10^{-1}\mu$mol/gDW [Bettenbrock et al., A quantitative approach to catabolite repression, JBC 281, 2006; J. Rohwer et al., Understanding glucose transport by the bacterial phosphoenolpyruvate: glucose phosphotransferase system on the basis of kinetic measurements *in vitro*, JBC 275, 2000] . Based on this values, a value of $r'_{fru} = 0.001$ was chosen.

For growth on CAA plus fructose, the complete set of equations has to be solved to determine the steady state degree of phosphorylation. The assumption of a steady state is reasonable since the phosphoryl transfer between the PTS proteins runs on a much faster time scale than metabolic reactions. Taking into account that some of the values for α_i are in the same order of magnitude, and with the values from above, the following set of equations is solved with MATLAB (.m files are available on request):

$$
\begin{aligned}
0 &= r'_1 - \frac{1}{100}\, r'_2 \\
0 &= r'_2 - r'_3 \\
0 &= r'_3 + r'_5 \\
0 &= r'_4 - \frac{1}{100}\, r'_5 - r'_{fru}
\end{aligned} \tag{4}
$$

Kinetic parameters

Based on the available data not all parameters could be calculated. Therefore, the following strategy was chosen: Parameter K_3 was first fixed to $K_3 = 1$. For growth in CAA the PEP/pyruvate ratio pp was set to 1. Considering the wild type, the conditions given in the main text and the available experimental data, the following condition that allows to determine K_1 holds true: The equations were written in such a form that the unknowns, $PtsP^P$ or $PtsO^P$, were eliminated:

$$
PtsP^P = \frac{K_2\, K_3}{1 - PtsP^P\,(1 - K_2\, K_3)} = \frac{pp}{K_1 + pp} \tag{5}
$$

Afterwards, the same equation is used to determine the PEP/pyruvate ratio pp for growth on CAA plus fructose. The product $K_4 \cdot K_5$ can be determined from the fact that for both branches, the equilibrium constant must be the same. Finally, considering growth on CAA plus fructose for the PtsP mutant, $r_3 = r_5 = 0$ and hence $r4' = r'_{fru}$ allows to calculate K_5. With the parameters so far, a value for K_2 was chosen in such a way that it fits the data at best. It was observed that there is a saturation behavior when varying K_2, that is, larger values of K_2 have no influence on the simulation results. Finally the influence of the

choice $K_3 = 1$ was analyzed. It turns out that K_3 could be changed only in a small range. Therefore $K_3 = 1$ was the final fixing. The resulting values are as follows (all values are dimensionless):

K_1	K_2	K_3	K_4	K_5
0.02	1.0	1.0	$3.12 \cdot 10^{-5}$	654.6

Figure 1 shows the influence of varying parameters K_2 and the PEP/pyruvate ratio since these values are set a priori. The default condition is marked with a black square. The colors represent values of the objective function (least square approach):

$$\Phi = \sum \frac{(y_{Mi} - y_i)^2}{\sigma^2} \qquad (6)$$

with y_{Mi} measured values, y_i simulated values and σ the standard deviation (values are given in Table 2 in the main text). The model can be assessed by comparing the values of Φ with values given from a χ^2 distribution. For the calculation shown in Figure 1, 10 data points are considered and five parameters are estimated (K_i and PEP/pyruvate ratio; note that not all K_i value could be chosen independently), so the range for χ^2 is given by 5 degree of freedom. The respective valid interval is therefore between 0.83 and 12.83.

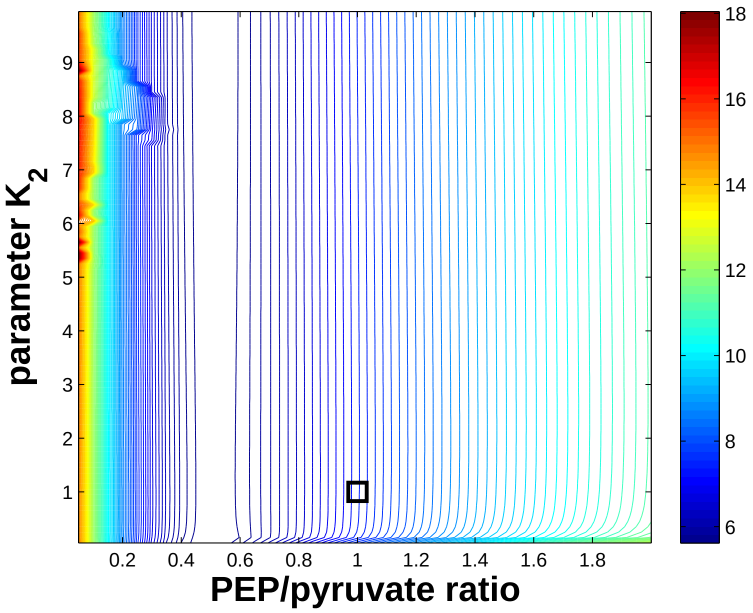

Figure 1: Contour plot for Φ in dependence on K_2 and the PEP/pyruvate ratio. The black square indicates the two values used in the calculations.

As can be seen, parameter K_2 has only marginal influence on the objective function. The PEP/pyruvate ratio could be changed in reasonable borders without leaving the interval given by the χ^2 distribution. The

black square indicates the two values used in the calculations.

Kinetics for the PEP synthase r_c and gluconeogenetic reaction r_d

Based on power law kinetics the following results are obtained for the two reactions:

$$
\begin{aligned}
r_c &= k_c\ Pyr^{n_c} \qquad \text{with} \qquad k_c = 1;\ n_c = 1 \quad \text{are set} \\
r_d &= k_d\ PEP^{n_d} \qquad \text{with} \qquad k_d = 2.73;\ n_d = 0.30
\end{aligned} \tag{7}
$$

Based on the values for the flux variability analysis, the value of k in Figure 8 might change. For the default condition $k = 0.34$. Since k is defined as $k = \frac{\Delta r_b}{\Delta r_a}$ it increases for larger values of Δr_b and smaller values of Δr_a and decreases for smaller values of Δr_b and larger values of Δr_a. Taking the values from Table 3 different values for Δr_a and Δr_b could be determined; the extreme conditions lead to values $k_{max} = 0.47$ and $k_{min} = 0.22$. These values are used in the calculation. The confidence region is shown in Figure 9 with dashed lines.
